# Combining a Pharmacological Network Model with a Bayesian Signal Detection Algorithm to Improve the Detection of Adverse Drug Events

**DOI:** 10.3389/fphar.2021.773135

**Published:** 2022-01-03

**Authors:** Xiangmin Ji, Guimei Cui, Chengzhen Xu, Jie Hou, Yunfei Zhang, Yan Ren

**Affiliations:** ^1^ School of Information Engineering, Inner Mongolia University of Science and Technology, Baotou, China; ^2^ School of Computer Science and Technology, Huaibei Normal University, Huaibei, China; ^3^ College of Intelligent Systems Science and Engineering, Harbin Engineering University, Harbin, China; ^4^ Department of Mathematics and Computer Engineering, Ordos Institute of Technology, Ordos, China

**Keywords:** adverse drug events, pharmacological network model, signal detection algorithm, FDA adverse event reporting system, pharmacovigilance

## Abstract

**Introduction:** Improving adverse drug event (ADE) detection is important for post-marketing drug safety surveillance. Existing statistical approaches can be further optimized owing to their high efficiency and low cost.

**Objective:** The objective of this study was to evaluate the proposed approach for use in pharmacovigilance, the early detection of potential ADEs, and the improvement of drug safety.

**Methods:** We developed a novel integrated approach, the Bayesian signal detection algorithm, based on the pharmacological network model (IC_PNM_) using the FDA Adverse Event Reporting System (FAERS) data published from 2004 to 2009 and from 2014 to 2019Q2, PubChem, and DrugBank database. First, we used a pharmacological network model to generate the probabilities for drug-ADE associations, which comprised the proper prior information component (IC). We then defined the probability of the propensity score adjustment based on a logistic regression model to control for the confounding bias. Finally, we chose the Side Effect Resource (SIDER) and the Observational Medical Outcomes Partnership (OMOP) data to evaluate the detection performance and robustness of the IC_PNM_ compared with the statistical approaches [disproportionality analysis (DPA)] by using the area under the receiver operator characteristics curve (AUC) and Youden’s index.

**Results:** Of the statistical approaches implemented, the IC_PNM_ showed the best performance (AUC, 0.8291; Youden’s index, 0.5836). Meanwhile, the AUCs of the IC, EBGM, ROR, and PRR were 0.7343, 0.7231, 0.6828, and 0.6721, respectively.

**Conclusion:** The proposed IC_PNM_ combined the strengths of the pharmacological network model and the Bayesian signal detection algorithm and performed better in detecting true drug-ADE associations. It also detected newer ADE signals than a DPA and may be complementary to the existing statistical approaches.

## Introduction

Adverse drug events (ADEs), which are unresolved and major issues in the medical field, pose a serious threat to public health. ADEs have resulted in high morbidity, mortality, and medical costs. In the United States, ADEs are the fourth leading cause of death after cancer and heart disease (Lazarou, et al., 1998) and cause more than 100,000 deaths per year (Giacomini, et al., 2007). Therefore, the early and accurate detection of potential ADEs can reduce health risks and improve drug safety. However, traditional toxicity testing and clinical trials are limited by issues such as sample sizes and the type of data collected in the pre-market stages, and risk management is continued in the post-market stages.

Improving the detection mechanism for ADEs is key to strengthening post-marketing drug safety surveillance (Harpaz, et al., 2017). Pharmacovigilance has been employed in the early detection of rare or unknown ADEs that were not found in the pre-market stages. Various computational methods have been developed and implemented using different databases that contain ADE information during the post-market stages. Among these databases, the US Food and Drug Administration’s Adverse Event Reporting System (FAERS) is one of the well-known spontaneous reporting systems (SRSs). A disproportionality analysis (DPA), also called a signal detection algorithm, is an important statistical approach used in the SRS analysis and is also used frequently to detect ADEs during pharmacovigilance. The proportional reporting ratio (PRR) and reporting odds ratio (ROR) are notable in frequentist DPAs (Evans et al., 2001; Van Puijenbroek, et al., 2002). The empirical Bayesian geometric mean (EBGM) and information component (IC) belong to the widely used Bayesian DPAs (Bate, et al., 1998; DuMouchel, 1999). Additionally, the three-component mixture model (3CMM) has been proposed based on the use of the EBGM, and the likelihood ratio test (LRT) as the frequentist method for the assessment of drug-ADE associations (Huang, et al., 2011; Zhang, et al., 2018). According to a recent study, among ten methods, IC achieved the best area under the receiver operator characteristics curve (AUC) (IC:0.6939) when OMOP is selected as the true ground for testing (Pham, et al., 2019). Another study showed that PRR and ROR had similar performances and that the EBGM outperformed the others (Harpaz, et al., 2013a). These findings were similar to those reported by Pham et al. Recently, a label propagation frame based on four popular signal detection algorithms (PRR, ROR, EBGM, IC) has emerged, which constructs a drug similarity network using chemical structures and combines pre-clinical drug chemical structures with the post-market database FAERS (Liu and Zhang, 2019). The different pharmacovigilance methods have been evaluated using a variety of performance metrics (Ding, et al., 2020).

However, DPA ignores the influence of a confounding bias in the analysis, which may lead to false positives and an under-detection of ADEs (DuMouchel, et al., 2013; Candore, et al., 2015). To overcome these limitations, machine learning algorithms and other methods have been used to detect ADEs using SRSs; some network-based methods and machine learning algorithms have been developed to predict ADEs using different public databases (Cami, et al., 2011; Liu, et al., 2012; Cheng, et al., 2013; Lin, et al., 2013; Davazdahemami and Delen, 2018; Jamal, et al., 2019). For example, a pharmacological network model (PNM) was developed to predict new and unknown drug-ADE associations (Cami, et al., 2011). The PNM integrated various types of data, including information from the Lexicomp, PubChem, and DrugBank databases. Phenotypic and chemical features based on the drug-ADE bipartite network were defined. Liu et al. integrated the phenotypic characteristics of drugs (i.e., indications and known adverse drug reactions), chemical structures, and biological properties of protein targets and pathway information, and used five machine learning methods to predict ADEs (Liu, et al., 2012). Moreover, Jamal et al. integrated the biological, chemical, and phenotypic features of drugs and used machine learning methods (random forest and sequential minimum optimization) to predict cardiovascular adverse reactions (Jamal, et al., 2019). Their results showed that the phenotypic data showed the best prediction effect and that drugs with similar chemical structures were more likely to exhibit similar ADEs. Furthermore, incorporating chemical and database information after marketing, which had the potential to detect clinically important ADEs.

Due to the rich value of DPAs in SRS analyses, we aimed to further optimize the use of DPAs and to improve ADE detection by combining the advantages of the Bayesian method and the pharmacological network model. In addition, the signal detection performances and properties of the top-ranked drug-ADE pairs generated by different DPAs were also investigated.

## Materials and Methods

### Study Scheme

**FIGURE 1:**
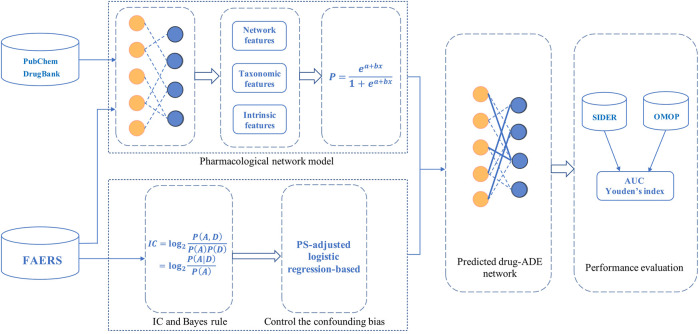
Overall framework, performance evaluation, and thus generating enhanced drug-ADE signals using a Bayesian signal detection algorithm based on pharmacological network model (IC_PNM_).

The overall framework of this study is shown in Figure 1. First, we constructed the drug-ADE network and trained the PNM using the FAERS, PubChem, and DrugBank databases. Second, the probabilities of drug-ADE associations (that not in the training data) were generated using the PNM. Third, IC was transformed using Bayes’ rule, and then the probability was predicted using the PNM as the prior probability in the IC algorithm after the Bayesian transformation. Finally, a Bayesian signal detection algorithm based on a pharmacological network model (IC_PNM_) was developed through data mining and the control of confounding biases based on a PS-adjusted logistic regression according to the data set.

### Data Sources

#### FAERS Database

As the largest SRS, the FAERS database collects ADE reports from physicians, manufacturers, nurses, and patients, and is updated quarterly (US Food and Drug Administration, 2020). We adopted a curated and standardized method to obtain FAERS data between 2004 and 2019Q2 (Banda, et al., 2016). For duplicated reports that shared the same primary IDs, the latest reports were used in our dataset. Drug names were mapped to RxNorm concepts, and the ADEs annotated in the Medical Dictionary for Regulatory Activities (MedDRA) were mapped to the preferred terms (PTs) (MedDRA, 2020). Herein, we selected two datasets as our sources to fully evaluate the performance and robustness, which included the FAERS data from 2004 to 2009 and the FAERS data from 2014 to 2019Q2.

FAERS 2004 data for the first 120 days were selected for the training set for the early detection of ADEs in the FAERS data between 2004 and 2009; the remaining FAERS data until 2009 were included in the testing set. All the drugs were extracted during this period and standardized according to the Drug Bank IDs (DrugBank, 2020). A total of 97 ADEs included the most concerning ADEs in the field of clinical medicine and the four ADEs from OMOP (these ADEs are listed in Supplementary Table S1). We then obtained 1,177 distinctive drugs, 107 to 97 ADEs, and 10,307 drug-ADE associations for the training set. The testing set included 22,358 new drug-ADE pairs (not in the training set) between 1,177 drugs and 97 ADEs. On the other hand, the FAERS data from 2014 to 2019Q2 were selected to further evaluate the performance and robustness of the proposed novel approach, in which FAERS 2014 data were chosen as the training set (3,500 drugs, 97 ADEs, and 27,821 drug-ADE associations) and the FAERS data from 2015 to 2019Q2 were chosen as the testing set (3,500 drugs and 97 ADEs, 24,300 drug-ADE associations that were not in the training set).

#### SIDER Database

The Side Effect Resource (SIDER) database contains marketed drugs and their recorded adverse drug reactions (ADRs), which are extracted from package inserts and public documents (Side Effect Resource, 2020). The current version 4.1 uses the MedDRA dictionary preferred terms; this dictionary contains 1,430 drugs, 5,868 ADRs, and 139,756 drug-ADE associations. We used the drug-ADE associations extracted from the SIDER 4.1 database as the true data for the analyses and evaluations. Among 22,358 drug-ADE pairs in the testing set, the intersection with the SIDER database revealed 1,148 pairs. Furthermore, among the 24,300 drug-ADE pairs in the testing set, the intersection with the SIDER database revealed 655 pairs.

#### OMOP Benchmark

The Observational Medical Outcomes Partnership (OMOP) established the gold standard for pharmacovigilance research (Ryan, et al., 2013). It contains 398 drug-ADE pairs composed of 181 drugs and four ADEs (acute myocardial infarction, acute renal failure, liver injury, and gastrointestinal bleeding), which were divided into 164 positive controls and 234 negative controls. We also used the OMOP gold standard to further evaluate the performance of the signal detection algorithms. Among the 22,358 drug-ADE pairs in the testing set, the intersection with the OMOP benchmark contained 158 pairs (80 positive controls and 78 negative controls). Moreover, among the 24,300 drug-ADE pairs in the testing set, the intersection with the OMOP benchmark contained 63 pairs (27 positive controls and 36 negative controls).

### Pharmacological Network Model

A pharmacological network model, also called a predictive pharmaco-safety network, was developed to predict new and unknown drug-ADE associations based on the drug-ADE bipartite network using FAERS, PubChem, and DrugBank database. The overview of PNM is shown in Supplementary Figure S1. The PNM generated three types of features, namely, network, taxonomic, and intrinsic features (14 of these features are listed in Supplementary Table S2). Based on these features, we trained a logistic regression (LR) model using the training data. In the LR model, the probabilities for drug-ADE associations being true were defined as follows:

$$p_{\mathrm{ij}}=\frac{\exp\left(\sum_{s}q_{s}x_{s}\left(i,j\right)\right)}{\left[1+\exp\left(\underset{s}{\sum }q_{s}x_{s}\left(i,j\right)\right)\right]}$$ (1)

Here, i denoted the number of drugs, j the number of ADEs, $q_{s}$ the regression parameter, $x_{s}$ the PNM features. We used the training data to fit the model through a 10-fold cross validation, and the optimal parameters were obtained using the optimal model that had the lowest Akaike Information Criterion (AIC).

Once we obtained a fully trained LR model, we could predict the probability of each drug-ADE association in the testing data using Eq. 2 as follows:

$$\mathrm{pr}o_{\mathrm{ij}}=\frac{1}{\left[1+\exp\left(-\underset{s}{\sum }q_{s}x_{s}\left(i,j\right)\right)\right]}$$ (2)

### Signal Detection Algorithms

Our research covered four classic signal detection algorithms: PRR, ROR, IC, and EBGM. Under the assumption that there was no association between the drug and the ADE, the DPAs assessed the drug-ADE associations by comparing the reported frequencies to the expected frequencies. We used the lower bound of the 95% confidence interval as the criterion for signal detection, and the main information of each algorithm is listed in Table 1. We defined $c_{ij}$ as the number of reports containing a drug-ADE pair. Furthermore, $c_{i+}$ and $c_{+j}$ were the number of reports containing the drug i and ADE j respectively, and $c_{++}$ was the total number of reports in the database.

**TABLE 1:** Association strength equation and threshold of signal detection algorithms.

	Method	Equation	Threshold
Frequentist statistical methods	PRR	$\mathrm{PRR}=\frac{c_{ij}/c_{i+}}{\left(c_{+j}-c_{ij}\right)/\left(c_{++}-c_{i+}\right)}$	PRR_025>1
	ROR	$\mathrm{ROR}=\frac{c_{ij}/\left(c_{+j}-c_{ij}\right)}{\left(c_{i+}-c_{ij}\right)/\left(c_{++}-c_{i+}-c_{+j}+c_{ij}\right)}$	ROR_025>1
Bayesian statistical methods	IC	$\mathrm{IC}∼{\text{log}}_{2}\frac{p_{ij}}{p_{i+}\times p_{+j}}$	IC_05>0
	EBGM	$\mathrm{EBG}M_{\mathrm{ij}}=2^{E\left[\text{log}\left(\lambda \right)|C=c_{ij}\right]/\text{log}\left(2\right)}$	EB_05≥2

### Bayesian Signal Detection Algorithm Based on the Pharmacological Network Model (IC_PNM_)

The IC is a measure of disproportionality in the Bayesian confidence propagation neural network (BCPNN). The IC assumes that the parameters follow the beta distribution to estimate the prior probability and assumes that the hyperparameter values are all 1. However, the PNM can generate probabilities for the drug-ADE associations, and these probabilities have different interpretations from the population the drug-ADE frequencies estimated using the SRS databases. To further improve ADE detection and optimize the statistical approach for pharmacovigilance, we developed a novel integrated method, namely, the Bayesian signal detection algorithm based on the pharmacological network model (IC_PNM_).

In our method, the Bayes rule gave the following transformation, and the IC was expressed as:

$$\mathrm{IC}=\mathrm{lo}g_{2}\frac{P\left(A,D\right)}{P\left(A\right)P\left(D\right)}=\mathrm{lo}g_{2}\frac{P\left(A|D\right)}{P\left(A\right)}$$ (3)

In this equation, (D) denoted the prior probability of a drug, which represented the probability of a drug appearing in the data set (A) the prior probability of an ADE, which represented the probability of an ADE appearing in the data set (A,D) the joint probability of the appearance of a drug and an ADE in the same report in the data set; and P(A|D) the conditional probability, which represented the probability of drug D inducing an ADE A.

As mentioned in Section 2.2, a PNM can generate probabilities for any drug-ADE association. Furthermore, the small sample size drug-ADE pairs contain more negative and positive data than large sample size drug-ADE pairs. In training of the PNM model, the training model should include both positive and negative data when selecting the training data (Ji, et al., 2021). Therefore, the PNM can control the influence of any confounding bias. The probability generated by the PNM was expressed as:

$$\mathrm{logit}\left[P\left(A_{j}|D_{i}\right)\right]=\alpha_{0}+\alpha_{1}x_{1}+\alpha_{2}x_{2}+\cdots \alpha_{14}x_{14}$$ (4)

Where, in Eq. 4, $\alpha_{0}$ , $\;\alpha_{1}\cdots \alpha_{14}$ denoted the regression parameters, and $\;x_{1},x_{2}\cdots x_{14}$ the 14 the features of the PNM.

Based on Eqs 3, 4, we combined the PNM and Bayesian methods and proposed an improved signal detection algorithm, called the Bayesian signal detection algorithm using the pharmacological network model (IC_PNM_), which was defined as follows:

$$IC_{\mathrm{PNM}}\left(i,j\right)=\mathrm{lo}g_{2}\frac{P\left(A_{j},D_{i}\right)}{P\left(A_{j}\right)P\left(D_{i}\right)}=\mathrm{lo}g_{2}\frac{P\left(A_{j}|D_{i}\right)}{P\left(A_{j}\right)}$$ (5)

Then, we calculated the probabilities of the ADEs in the dataset. When the data contained sufficient independent and identically distributed samples, the probability of an ADE $A_{j}$ was obtained using the frequency value $Pr\left(A\right)=c_{+j}/c_{++}\;$ from the data set according to Bernoulli’s law of large numbers. Pr(A) was also rewritten according to Eq. 6. However, this probability did not consider the influence of any confounding bias.

$$\mathrm{Pr}\left(A_{j}\right)=\frac{\exp\left(\beta \right)}{1+\exp\left(\beta \right)}$$ (6)

Where, in Equation 6, $\beta =\text{ln}\frac{C_{+j}/C_{++}}{1-C_{+j}/C_{++}}$ .

When using FAERS data to detect ADE signals, the confounding factors in the data may affect the results and cause signal masking or result in false positive signals. The common confounding factors in FAERS include the patient demographics (such as age, gender, etc., as these data contribute greatly to missing data), combined medication information, etc., among which combined medication is a common phenomenon in the data. To eliminate the influence of combined medication on the results and correct for the confounding bias caused due to it, we calculated the propensity score (PS) to address the confounding bias in the data set. The confounding bias caused by combined medication is the propensity score of drugs, which represented the probability of drug exposure in each report. In other words, the probability of drug selection (that is, the propensity score of each drug) was computed using Eq. 7. Subsequently, for each drug-ADE pair, we estimated the drug effect with an adjustment of PS through the logistic regression model (8).

$$\mathrm{Logit}\left[P\left(\mathrm{Drug}=1\right)\right]=\gamma_{0}+\overset{n}{\underset{i=1}{\sum }}\gamma_{i}PC_{i}$$ (7)

$$\mathrm{Logit}\left[P\left(\mathrm{ADE}=1\right)\right]=\beta_{0}+\beta_{1}\times \mathrm{Drug}+\beta_{2}\times \mathrm{PS}$$ (8)

In Equation 7, n denoted the number of principle components (PCs). From Eq. 8, we proposed and defined the probability $P\left(A_{j}\right)$ of ADE according to the PS-adjusted logistic regression. Assuming the estimated value of the regression coefficient in Eq. 8 as $\left(\tilde{\beta_{0}},\tilde{\beta_{1}},\tilde{\beta_{2}}\right)$ , for K reports, $P\left(A_{j}\right)$ was expressed as follows with an adjustment for PS value:

$$P\left(A_{j}\right)=\overset{k}{\underset{k=1}{\sum }}\frac{\exp\left(\tilde{\beta }+\tilde{\beta_{0}}+\tilde{\beta_{1}}+\tilde{\beta_{2}}PS_{k}\right)}{1+\exp\left(\tilde{\beta }+\tilde{\beta_{0}}+\tilde{\beta_{1}}+\tilde{\beta_{2}}PS_{k}\right)}$$ (9)

According to Eqs 4, 5, 9, the lower bound of the 95% confidence interval (IC_PNM__05) for IC_PNM_ was defined as follows, and the criterion for signal detection was an IC_PNM__05 of ≥ 2. This criterion ensured with a high degree of confidence that, regardless of the count size, the frequency of reporting drug-ADE association was at least twice that when there was no association between the drug and ADE.

$$IC_{\mathrm{PNM}}\_05=\mathrm{lo}g_{2}\frac{P\left(A_{j}|D_{i}\right)}{P\left(A_{j}\right)}\cdot \exp\left(\frac{-2}{\sqrt{c_{\mathrm{ij}}+1}}\right)$$ (10)

In Equation 10, $P\left(A_{j}|D_{i}\right)$ is generated by PNM, and $P\left(A_{j}\right)$ is obtained from Eq. 9.

## Results

### Evaluation and Comparison With Other Signal Detection Algorithms

**FIGURE 2:**
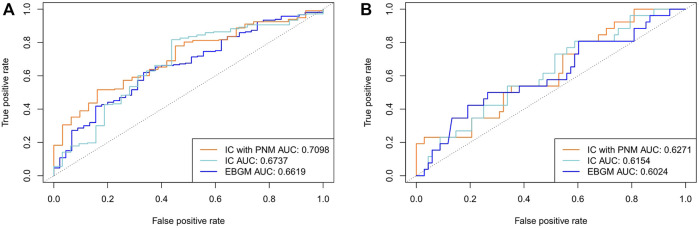
**(A)** Comparison of performances of IC_PNM_, IC, and EBGM with SIDER data as the testing set using FAERS 2004–2009 data **(B)** Comparison of performances of IC_PNM_, IC, and EBGM with OMOP data as the testing set using FAERS 2004–2009 data.

We compared the proposed IC_PNM_ method with the Bayesian statistical methods (IC and EBGM) using 1,148 pairs that intersected with the SIDER database and 158 pairs that intersected with the OMOP benchmark, respectively, as the testing set from FAERS between 2004 and 2009. The detection performance is presented in Figure 2. The IC_PNM_ performed better than the IC and EBGM in terms of AUC scores when 1,148 SIDER drug-ADE pairs were used as the testing set (AUC scores: 0.7098, 0.6737, and 0.6619 for IC_PNM_, IC, and EBGM, respectively). Furthermore, IC_PNM_ still achieved better performance when 158 OMOP drug-ADE pairs were the testing set (AUC scores: 0.6271, 0.6154, and 0.6024 for IC_PNM_, IC, and EBGM, respectively). EBGM showed a generally worse performance compared with the other two methods. On the other hand, from the ROC for three Bayesian statistical methods plotted in Figure 2A, IC_PNM_ had higher sensitivity at high specificity points (>0.6 specificity), whereas IC had higher sensitivity at low specificity points (0.3<specificity<0.6), followed by IC_PNM_. In contrast, we performed quantitative bias analysis when IC_PNM_ did not control the confounding bias, and the confusion matrices were listed in Supplementary Tables S3A, S4A. In summary, the above results presented that the IC_PNM_ can enhance drug safety because it combined the strengths of both the PNM and the Bayesian methods and it controlled the confounding bias.

**FIGURE 3:**
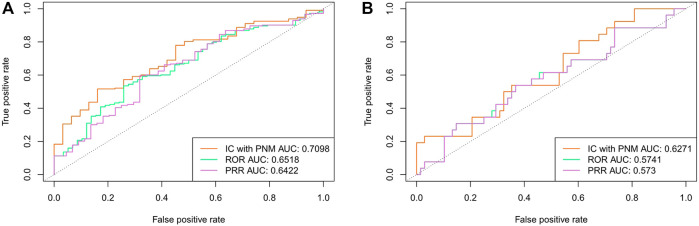
**(A)** Comparison of performances of IC_PNM_, ROR, and PRR with SIDER data as the testing set using FAERS 2004–2009 data **(B)** Comparison of performances of IC_PNM_, ROR, and PRR with OMOP data as the testing set using FAERS 2004–2009 data.

We also evaluated the performance of the IC_PNM_ compared with the frequentist statistical methods (ROR and PRR) using the FAERS data between 2004 and 2009. As shown in Figure 3, IC_PNM_ still performed better than ROR and PRR in terms of AUC scores when 1,148 drug-ADE pairs and 158 drug-ADE pairs were the testing set, respectively. In general, the Bayesian statistical methods were superior to the frequentist methods, and among them, the ROR performed better than the PRR.

Overall, AUCs, Youden’s sensitivities and Youden’s specificities and Youden’s indices for our IC_PNM_ and other four statistical methods are shown in Table 2, which were calculated using the FAERS data between 2004 and 2009 based on SIDER and OMOP. The maximum of sensitivity and specificity values are Youden’s index, and the position of the Youden index indicates the optimal cut-off point of an algorithm’s decision threshold. When SIDER data was used as the testing set, the IC_PNM_ had the highest AUC and Youden’s index, and the IC had the second highest. When the OMOP data was used as the testing set, the IC_PNM_ still had the highest AUC and Youden’s index, and IC the second-highest AUC and third-highest Youden’s index. Moreover, EBGM had the third-highest AUC and second-highest Youden’s index. The confusion matrices for the results of Table 2 are presented in Supplementary Tables S3, S4.

**TABLE 2:** Results of performance of different signal detection algorithms using FAERS 2004–2009 data.

Testing set	Method	AUC	Youden’s sensitivity	Youden’s specificity	Youden’s index
SIDER	IC_PNM_	**0.7098**	0.5264	0.8547	**0.3811**
	IC	0.6737	0.8169	0.5625	0.3794
	EBGM	0.6619	0.6297	0.6667	0.2964
	ROR	0.6518	0.5352	0.7414	0.2766
	PRR	0.6422	0.5963	0.6818	0.2781
OMOP	IC_PNM_	**0.6271**	0.5284	0.7251	**0.2535**
	IC	0.6154	0.8077	0.4118	0.2195
	EBGM	0.6024	0.5012	0.7353	0.2365
	ROR	0.5741	0.5385	0.6324	0.1709
	PRR	0.5730	0.5385	0.6324	0.1709

The bold values are to highlight the performance of the method, and have no specific meaning.

### Evaluating the Performance and Robustness of IC_PNM_

**FIGURE 4:**
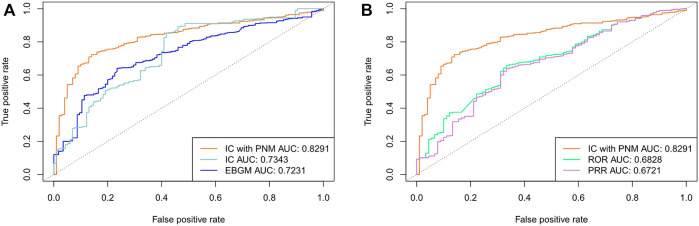
**(A)** Comparison of performances of IC_PNM_, IC, and EBGM with SIDER data as the testing set using FAERS 2014-2019Q2 data **(B)** Comparison of performances of IC_PNM_, ROR, and PRR with SIDER data as the testing set using FAERS 2014-2019Q2 data.

To verify the performance and robustness of the proposed method, we selected the FAERS data from 2014 to 2019Q2 and the SIDER data for additional analyses. The detection performance is shown in Figure 4, and the experimental results are summarized in Table 3. Among the five signal detection algorithms, the IC_PNM_ had the highest performance (AUC score,0.8291; Youden’s index, 0.5836) for these statistical methods. On the other hand, from the ROC for Bayesian methods and frequentist methods plotted in Figure 4, IC_PNM_ still had higher sensitivity at high specificity points (>0.6 specificity), whereas IC_PNM_’s sensitivity was close to the sensitivity of IC at low specificity points (specificity<0.6), and higher than that of EBGM. It is further confirmed that Bayesian DPAs were superior to frequentist DPAs. Our experiments also showed that the signals generated using our cut-off have high enough specificity to deserve further investigation. In contrast, we also performed quantitative bias analysis when IC_PNM_ did not control the confounding bias, and the confusion matrices were listed in Supplementary Table S5A. The result showed that controlling the confounding bias could improve the performance of the algorithm. Moreover, the confusion matrices for the results of Table 3 are presented in Supplementary Table S5.

**TABLE 3:** Results of performance of different signal detection algorithms using FAERS 2014–2019Q2 data.

Testing set	Method	AUC	Youden’s sensitivity	Youden’s specificity	Youden’s index
SIDER	IC_PNM_	**0.8291**	0.7236	0.8600	**0.5836**
	IC	0.7343	0.8537	0.5826	0.4363
	EBGM	0.7231	0.6407	0.7652	0.4059
	ROR	0.6828	0.6561	0.6667	0.3228
	PRR	0.6721	0.6381	0.6667	0.3048

The bold values are to highlight the performance of the method, and have no specific meaning.

**FIGURE 5:**
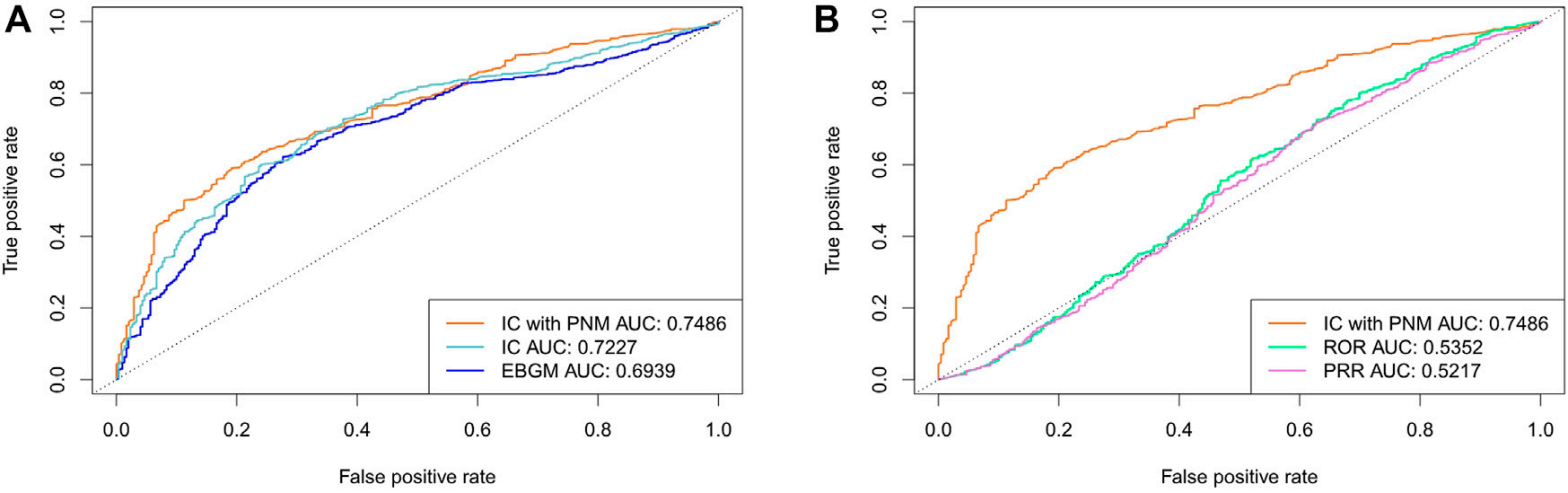
**(A)** Comparison of performances of IC_PNM_, IC, and EBGM using cross validation based on the FAERS 2014-2019Q2 data and SIDER **(B)** Comparison of performances of IC_PNM_, ROR, and PRR using cross validation based on the FAERS 2014-2019Q2 data and SIDER.

We further evaluated the performance using cross validation and the training set composed of 10% of the data from each year (2014-2019Q2). The detection performance is shown in Table 4 and Figure 5. Among the five signal detection algorithms, IC_PNM_ had the highest performance (AUC score, 0.7486; Youden’s index, 0.3993) for these methods. Furthermore, from the ROC for Bayesian methods and frequentist methods plotted in Figure 5, IC_PNM_ still had higher sensitivity at high specificity points (>0.6 specificity), whereas IC_PNM_’s sensitivity was higher than the sensitivity of IC at low specificity points (specificity<0.6), and higher than that of EBGM.

**TABLE 4:** Results of performance of different signal detection algorithms using cross validation and FAERS 2014–2019Q2 data.

Testing set	Method	AUC	Youden’s sensitivity	Youden’s specificity	Youden’s index
SIDER	IC_PNM_	**0.7486**	0.5909	0.8083	**0.3992**
	IC	0.7227	0.7983	0.3233	0.1216
	EBGM	0.6939	0.6222	0.7233	0.3455
	ROR	0.5352	0.7999	0.3010	0.1009
	PRR	0.5217	0.7171	0.3733	0.0904

The bold values are to highlight the performance of the method, and have no specific meaning.

For the IC_PNM_, we further performed analysis using different thresholds and compared the results. Table 5 provided performance metrics for sensitivity, specificity, and different threshold values. When the threshold decreased, sensitivity increased, specificity decreased and PPV also decreased. For example, when the threshold was decreased to 1, the sensitivity increased to 0.9642 at the expense of dropping specificity to 0.1333. In contrast, when the threshold increased to 3, sensitivity decreased to 0.6325, specificity increased to 0.91, and the PPV was 0.9773.

**TABLE 5:** Performance metrics of IC_PNM_ based on the different threshold values, sensitivity, specificity and PPV using FAERS 2014–2019Q2 data.

Threshold	IC_PNM_		
	Sensitivity	Specificity	PPV
1	0.9642	0.1333	0.8202
1.5	0.9138	0.36	0.8541
2	0.8617	0.54	0.8848
2.5	0.756	0.8	0.9394
3	0.6325	0.91	0.9773

PPV, positive predictive value.

We analyzed the correlation between the proposed IC_PNM_ algorithm and the other statistical methods (IC, EBGM, ROR, and PRR). The correlation coefficients of the IC_PNM_ with the EBGM and IC were 0.6619 and 0.5039, respectively, and the correlation coefficients with the ROR and the PRR were 0.1606 and 0.1602, respectively (Detailed information is presented in Supplementary Table S6). According to these results, IC_PNM_ not only had a superior performance but also complemented the existing statistical approaches.

### Properties of the Top-Ranked Signals

We observed the top 50 signals generated using different statistical approaches. The Top-50 drug-ADE signals were further investigated using the SIDER data. First, the FAERS data between 2004 and 2009 were selected as the testing data. For the frequentist statistical methods, none of the top 50-ranked signals identified by ROR and PRR were validated using the SIDER data. For the Bayesian statistical methods, 13 of the top 50-ranked signals identified by the EBGM were validated using the SIDER data, and the IC_PNM_ and the IC had 10 and 9 drug-ADE pairs, respectively. Among the 13 signals of the EBGM and the nine signals of the IC, the four signals were the same. Ten IC_PNM_ signals were completely different from those of the EBGM and the IC. Next, the FAERS data between 2014 and 2019Q2 were selected as the testing data. For the frequentist statistical methods, one of the top 50-ranked signals identified by the ROR were validated using the SIDER data, and one using the PRR, with the overlapping signal being the same. For the Bayesian statistical methods, the IC_PNM_, IC, and EBGM had 12, 9, and 12 overlapping drug-ADE signals using the SIDER data, respectively. Among the 12 signals of the EBGM and the nine signals of the IC, seven signals were the same. Among the 12 signals of the EBGM and the 12 signals of the IC_PNM_, four signals were the same. There were two overlapping signals between the IC_PNM_ and the IC (Signals identified by each approach using the SIDER data are shown in Supplementary Tables S7, S8).

## Discussion

This study was designed to evaluate the performance of statistical methods in detecting unknown and new drug safety signals early and accurately. The performance of our proposed approach IC_PNM_ was superior to that of a DPA using AUC and Youden’s index. Furthermore, IC_PNM_ performed well on the high and low ends of specificity and had the highest sensitivity among the DPAs when specificity was >0.6, here using 1,177 drugs and 97 ADEs in FAERS 2004-2009 as the experimental data. This also meant that IC_PNM_ had good performance in detecting true-positive signals and false-positive signals. Then IC_PNM_ had a higher sensitivity and specificity using 3,500 drugs and 97 ADEs in FAERS 2014-2019Q2 data. We believe that the increase in the number of drugs in the training data can improve the performance of the algorithm. In some cases, the traditional DPA performed well and was simple to calculate. However, the lack of accuracy in the signal detection, which may have been influenced by noise, may have caused important signals to be missed and the inclusion of some false-positive signals. These limitations may be attributed to the characteristics of these methods. At the same time, although SRSs also suffered from some limitations due to their own attributes, such as the overreporting and misattribution of causality, SRSs still have the advantage of being irreplaceable in drug safety surveillance (Harpaz, et al., 2012).

To improve ADE detection and overcome the limitations with the use of traditional DPAs, the signal detection algorithm IC_PNM_ was developed based on different types of databases. Meanwhile, several other related studies confirmed that the utilization and combination of multiple databases could improve the detection of ADEs (Harpaz, et al., 2012; Xu and Wang, 2014; Li, et al., 2015; Harpaz, et al., 2017; Li, et al., 2020). However, the publicly available FAERS database required data curation before it could be used correctly, and different data cleaning and standardization strategies may have had a significant impact on the analysis results. Therefore, the first step was to process the FARES data. We used a curated and standardized method to obtain the data (Banda, et al., 2016). Then, using the FAERS, PubChem, and DrugBank databases, we extracted various types of information. The IC_PNM_ calculated the probabilities for the drug-ADE associations using the network, taxonomic, and intrinsic features based on the PNM. Among the algorithms tested, the IC_PNM_ achieved the best performance in detecting true signals while controlling for any confounding bias. The IC_PNM_ performed well in ADE detection, and the AUCs of previous similar studies using the OMOP Benchmark 398 drug-ADE pairs was less than 0.75 (Zhang, et al., 2018; Pham, et al., 2019; Harpaz, et al., 2013b).

The performance of the proposed algorithm can be explained by several important strengths. First, the IC_PNM_ used the chemical and phenotypic characteristics and logistical regression models to calculate the probability of drug-ADE pairs. The IC_PNM_ combined pre-clinical drug information with post-marketing safety reports. Furthermore, while the drug-ADE pairs with small sample sizes had more negative data than the drug-ADE pairs with large sample sizes, they also contained positive data. As stated in our previous research, in training a PNM model, the training model should include both large and small sample size drug-ADE pairs when selecting the training data (Ji, et al., 2021). Therefore, the probabilities calculated by PNM were not influenced by any confounders. Second, the IC_PNM_ calculated the defined feature effects based on a drug-ADE bipartite network, which effectively reflected the properties of the drugs and made use of a powerful network function compared to the DPA. Finally, we proposed and defined a PS-adjusted logistic regression based on the control of the confounding bias from the FAERS data. In contrast, the influence of the confounding bias was ignored in the DPA analysis, which may have led to a signal bias and hence, inaccuracy. The IC_PNM_ generated enhanced safety signals through the probability generated by PNM as the prior probability and PS-adjusted logistic regression.

Our proposed IC_PNM_ detected potential ADE signals that were not detected by the traditional DPAs. It was challenging to identify the different ADEs using limited data. Hence, it is important to be able to detect potential ADEs using a post-market database. The traditional DPAs (IC, EBGM, ROR and PRR) showed insufficient sensitivities or specificities, resulting in false-negative or false-positive results, and their advantages and disadvantages were discussed in the recent scientific literature (Ding, et al., 2020). An important finding of our study was that the top signals of the different signal detection algorithms had different patterns. For instance, the top-50 signals generated by the IC_PNM_ and the DPAs, the IC and the EBGM had the most overlapped signals while the IC_PNM_ had fewer signals due to its powerfully different patterns. These results demonstrated that the signals generated by IC_PNM_ were complementary to the existing statistical methods. This study also showed that the use of a combination of different signal detection algorithms in quantitative detection research achieved higher accuracy compared with the use of a signal detection algorithm alone.

Our study had some limitations. First, the performance of the proposed IC_PNM_ relied heavily on the features of the PNM and the training data. Among them, the network features of the PNM had obvious advantages; the taxonomic and intrinsic features improved the prediction performance; however, they also increased the complexity of the data. To improve the practicability of the IC_PNM_ to SRS alone, the use of network features only can also generate probabilities for the drug-ADE associations. Second, while the variety of the confounding variables can be controlled using multiple regression or propensity score analyses (Caster, et al., 2010; Tatonetti, et al., 2012; Tatonetti, et al., 2011), it was not easy to integrate the confounding variables into Bayesian DPA methods (especially, patient demographic information such as age, gender, etc., which have a large number of missing data in FAERS database), and the development of an appropriate methodology was required (Goldstein, et al., 2017). Currently, there is no signal detection algorithm that can overcome all such data quality problems. Third, as discussed in the literature by Ding et al., other limitations of these DPAs include relying on subjective thresholds. Youden index can be used as a comprehensive index to evaluate the ability of methods. In the future, further research is needed on how to select the optimal threshold without affecting the sensitivity. Lastly, we used the SIDER database and OMOP benchmarks as the gold standards against which to evaluate the performance of the IC_PNM_ and DPAs. Using different databases and reference sets might lead to different performance characteristics.

In conclusion, our novel Bayesian signal detection algorithm, the IC_PNM_, which combined a pharmacological network model with the Bayesian method, achieved superior performance and detected newer ADE signals compared with that achieved with the use of traditional DPAs. The use of the IC_PNM_ generated drug safety signals using data from the post-market database FAERS and pre-clinical drug information, and it controlled the confounding bias using a PS-adjusted logistic regression. Additionally, an increase in the number of drugs in the training set can improve the performance of the algorithm, that is, IC_PNM_ can obtain superior AUC, specificity, and sensitivity. Moreover, the signals generated using different methods had different patterns, and they complemented each other. Thus, the IC_PNM_ not only had a better performance but also complemented the existing statistical approaches.

## Data Availability

The original contributions presented in the study are included in the article/Supplementary Material, further inquiries can be directed to the corresponding authors.

## References

[B1] Banda J. M. Evans L. Vanguri R. S. Tatonetti N. P. Ryan P. B. Shah N. H. (2016). A Curated and Standardized Adverse Drug Event Resource to Accelerate Drug Safety Research. Sci. Data 3, 160026. 10.1038/sdata.2016.26 27193236 PMC4872271

[B2] Bate A. Lindquist M. Edwards I. R. Olsson S. Orre R. Lansner A. (1998). A Bayesian Neural Network Method for Adverse Drug Reaction Signal Generation. Eur. J. Clin. Pharmacol. 54, 315–321. 10.1007/s002280050466 9696956

[B3] Cami A. Arnold A. Manzi S. Reis B. (2011). Predicting Adverse Drug Events Using Pharmacological Network Models. Sci. Transl Med. 3, 114ra127–127. 10.1126/scitranslmed.3002774 22190238

[B4] Candore G. Juhlin K. Manlik K. Thakrar B. Quarcoo N. Seabroke S. (2015). Comparison of Statistical Signal Detection Methods within and across Spontaneous Reporting Databases. Drug Saf. 38, 577–587. 10.1007/s40264-015-0289-5 25899605

[B5] Caster O. Norén G. N. Madigan D. Bate A. (2010). Large-scale Regression-Based Pattern Discovery: The Example of Screening the WHO Global Drug Safety Database. Stat. Analy Data Mining 3, 197–208. 10.1002/sam.10078

[B6] Cheng F. Li W. Wang X. Zhou Y. Wu Z. Shen J. (2013). Adverse Drug Events: Database Construction and In Silico Prediction. J. Chem. Inf. Model. 53, 744–752. 10.1021/ci4000079 23521697

[B7] Davazdahemami B. Delen D. (2018). A Chronological Pharmacovigilance Network Analytics Approach for Predicting Adverse Drug Events. J. Am. Med. Inform. Assoc. 25, 1311–1321. 10.1093/jamia/ocy097 30085102 PMC7646912

[B8] Ding Y. Markatou M. Ball R. (2020). An Evaluation of Statistical Approaches to Postmarketing Surveillance. Stat. Med. 39, 845–874. 10.1002/sim.8447 31912927

[B9] DrugBank (2020). Available From: https://go.drugbank.com .

[B10] DuMouchel W. Ryan P. B. Schuemie M. J. Madigan D. (2013). Evaluation of Disproportionality Safety Signaling Applied to Healthcare Databases. Drug Saf. 36, S123–S132. 10.1007/s40264-013-0106-y 24166229

[B11] DuMouchel W. (1999). Bayesian Data Mining in Large Frequency Tables, with an Application to the FDA Spontaneous Reporting System. The Am. Statistician 53, 177–190. 10.1080/00031305.1999.10474456

[B12] Evans S. J. Waller P. C. Davis S. (2001). Use of Proportional Reporting Ratios (PRRs) for Signal Generation from Spontaneous Adverse Drug Reaction Reports. Pharmacoepidemiol. Drug Saf. 10, 483–486. 10.1002/pds.677 11828828

[B13] Giacomini K. M. Krauss R. M. Roden D. M. Eichelbaum M. Hayden M. R. Nakamura Y. (2007). When Good Drugs Go Bad. Nature 446, 975–977. 10.1038/446975a 17460642

[B14] Goldstein B. A. Navar A. M. Pencina M. J. Ioannidis J. P. (2017). Opportunities and Challenges in Developing Risk Prediction Models with Electronic Health Records Data: a Systematic Review. J. Am. Med. Inform. Assoc. 24, 198–208. 10.1093/jamia/ocw042 27189013 PMC5201180

[B15] Harpaz R. Dumouchel W. Lependu P. Bauer-Mehren A. Ryan P. Shah N. H. (2013a). Performance of Pharmacovigilance Signal-Detection Algorithms for the FDA Adverse Event Reporting System. Clin. Pharmacol. Ther. 93, 539–546. 10.1038/clpt.2013.24 23571771 PMC3857139

[B16] Harpaz R. Dumouchel W. Schuemie M. Bodenreider O. Friedman C. Horvitz E. (2017). Toward Multimodal Signal Detection of Adverse Drug Reactions. J. Biomed. Inform. 76, 41–49. 10.1016/j.jbi.2017.10.013 29081385 PMC8502488

[B17] Harpaz R. Dumouchel W. Shah N. H. Madigan D. Ryan P. Friedman C. (2012). Novel Data-Mining Methodologies for Adverse Drug Event Discovery and Analysis. Clin. Pharmacol. Ther. 91, 1010–1021. 10.1038/clpt.2012.50 22549283 PMC3675775

[B18] Harpaz R. Vilar S. DuMouchel W. Salmasian H. Haerian K. Shah N. H. (2013b). Combing Signals from Spontaneous Reports and Electronic Health Records for Detection of Adverse Drug Reactions. J. Am. Med. Inform. Assoc. 20, 413–419. 10.1136/amiajnl-2012-000930 23118093 PMC3628045

[B19] Huang L. Zalkikar J. Tiwari R. C. (2011). A Likelihood Ratio Test Based Method for Signal Detection with Application to FDA's Drug Safety Data. J. Am. Stat. Assoc. 106, 1230–1241. 10.1198/jasa.2011.ap10243

[B20] Jamal S. Ali W. Nagpal P. Grover S. Grover A. (2019). Computational Models for the Prediction of Adverse Cardiovascular Drug Reactions. J. Transl Med. 17, 171. 10.1186/s12967-019-1918-z 31118067 PMC6530172

[B21] Ji X. Wang L. Hua L. Wang X. Zhang P. Shendre A. (2021). Improved Adverse Drug Event Prediction through Information Component Guided Pharmacological Network Model (IC-PNM). Ieee/acm Trans. Comput. Biol. Bioinf. 18, 1113–1121. 10.1109/TCBB.2019.2928305 31443040

[B22] Lazarou J. Pomeranz B. H. Corey P. N. (1998). Incidence of Adverse Drug Reactions in Hospitalized Patients: a Meta-Analysis of Prospective Studies. JAMA 279, 1200–1205. 10.1001/jama.279.15.1200 9555760

[B23] Li Y. Jimeno Yepes A. Xiao C. (2020). Combining Social media and FDA Adverse Event Reporting System to Detect Adverse Drug Reactions. Drug Saf. 43, 893–903. 10.1007/s40264-020-00943-2 32385840 PMC7434724

[B24] Li Y. Ryan P. B. Wei Y. Friedman C. (2015). A Method to Combine Signals from Spontaneous Reporting Systems and Observational Healthcare Data to Detect Adverse Drug Reactions. Drug Saf. 38, 895–908. 10.1007/s40264-015-0314-8 26153397 PMC4579260

[B25] Lin J. Kuang Q. Li Y. Zhang Y. Sun J. Ding Z. (2013). Prediction of Adverse Drug Reactions by a Network Based External Link Prediction Method. Anal. Methods 5, 6120–6127. 10.1039/c3ay41290c

[B26] Liu M. Wu Y. Chen Y. Sun J. Zhao Z. Chen X. W. (2012). Large-scale Prediction of Adverse Drug Reactions Using Chemical, Biological, and Phenotypic Properties of Drugs. J. Am. Med. Inform. Assoc. 19, e28–35. 10.1136/amiajnl-2011-000699 22718037 PMC3392844

[B27] Liu R. Zhang P. (2019). Towards Early Detection of Adverse Drug Reactions: Combining Pre-clinical Drug Structures and post-market Safety Reports. BMC Med. Inform. Decis. Mak 19, 279. 10.1186/s12911-019-0999-1 31849321 PMC6918608

[B28] MedDRA (2020). Available From: http://www.meddramsso.com/index.asp .

[B29] Pham M. Cheng F. Ramachandran K. (2019). A Comparison Study of Algorithms to Detect Drug-Adverse Event Associations: Frequentist, Bayesian, and Machine-Learning Approaches. Drug Saf. 42, 743–750. 10.1007/s40264-018-00792-0 30762164

[B30] Ryan P. B. Schuemie M. J. Welebob E. Duke J. Valentine S. Hartzema A. G. (2013). Defining a Reference Set to Support Methodological Research in Drug Safety. Drug Saf. 36, S33–S47. 10.1007/s40264-013-0097-8 24166222

[B31] Side Effect Resource (2020). Available From: http://sideeffects.embl.de/

[B32] Tatonetti N. P. Denny J. C. Murphy S. N. Fernald G. H. Krishnan G. Castro V. (2011). Detecting Drug Interactions from Adverse-Event Reports: Interaction between Paroxetine and Pravastatin Increases Blood Glucose Levels. Clin. Pharmacol. Ther. 90, 133–142. 10.1038/clpt.2011.83 21613990 PMC3216673

[B33] Tatonetti N. P. Ye P. P. Daneshjou R. Altman R. B. (2012). Data-driven Prediction of Drug Effects and Interactions. Sci. Transl. Med. 4, 125ra31–131. 10.1126/scitranslmed.3003377 PMC3382018 22422992

[B34] Us Food and Drug Administration (2020). Adverse Event Reporting System (FAERS). Available From: https://www.fda.gov/Drugs/InformationOnDrugs/ucm135151.htm .

[B35] Van Puijenbroek E. P. Bate A. Leufkens H. G. Lindquist M. Orre R. Egberts A. C. (2002). A Comparison of Measures of Disproportionality for Signal Detection in Spontaneous Reporting Systems for Adverse Drug Reactions. Pharmacoepidemiol. Drug Saf. 11, 3–10. 10.1002/pds.668 11998548

[B36] Xu R. Wang Q. (2014). Large-scale Combining Signals from Both Biomedical Literature and the FDA Adverse Event Reporting System (FAERS) to Improve post-marketing Drug Safety Signal Detection. BMC Bioinformatics 15, 17. 10.1186/1471-2105-15-17 24428898 PMC3906761

[B37] Zhang P. Li M. Chiang C. W. Wang L. Xiang Y. Cheng L. (2018). Three-Component Mixture Model-Based Adverse Drug Event Signal Detection for the Adverse Event Reporting System. CPT Pharmacometrics Syst. Pharmacol. 7, 499–506. 10.1002/psp4.12294 30091855 PMC6118321

