# Supplementary material for: Combining a Pharmacological Network Model with a Bayesian Signal Detection Algorithm to Improve the Detection of Adverse Drug Events
Source: Front Pharmacol. 2022 Jan 3;12:773135. doi: 10.3389/fphar.2021.773135 (PMC8762263; doi:10.3389/fphar.2021.773135)
Supplement: Supplementary file 1 [file DataSheet1.docx]

Supplementary Material

# Supplementary Tables

**Table S1** List of 97 ADEs and their PT codes

| ADE name | PT code |
| --- | --- |
| Acute hepatic failure | 10000804 |
| acute myocardial infarction | 10000891 |
| Asterixis | 10003547 |
| Axonal neuropathy | 10003882 |
| Biopsy liver abnormal | 10004792 |
| Cholestasis | 10008635 |
| Coma hepatic | 10010075 |
| Corneal deposits | 10011000 |
| Corneal opacity | 10011035 |
| Delirium | 10012218 |
| Diabetic autonomic neuropathy | 10012645 |
| Diabetic mononeuropathy | 10012676 |
| Diabetic neuropathy | 10012680 |
| Fatty liver alcoholic | 10016262 |
| Gastrointestinal haemorrhage | 10017955 |
| Granulomatous liver disease | 10018704 |
| Hepatic cirrhosis | 10019641 |
| Hepatic encephalopathy | 10019660 |
| Hepatic failure | 10019663 |
| Hepatic function abnormal | 10019670 |
| Hepatic necrosis | 10019692 |
| Hepatitis cholestatic | 10019754 |
| Hepatocellular injury | 10019837 |
| Hepatomegaly | 10019842 |
| Hepatorenal failure | 10019845 |
| Hepatorenal syndrome | 10019846 |
| Histiocytosis | 10020117 |
| Hyperbilirubinaemia | 10020578 |
| Hyperbilirubinaemia neonatal | 10020580 |
| Jaundice | 10023126 |
| Jaundice acholuric | 10023128 |
| Jaundice cholestatic | 10023129 |
| Jaundice extrahepatic obstructive | 10023130 |
| Kernicterus | 10023376 |
| Lipidosis | 10024585 |
| Liver function test abnormal | 10024690 |
| Lung infiltration | 10025102 |
| Lupoid hepatic cirrhosis | 10025129 |
| Lymphadenopathy | 10025197 |
| Macrophages decreased | 10025392 |
| Mononeuropathy multiplex | 10027918 |
| Muscle injury | 10028314 |
| Muscle spasms | 10028334 |
| Muscular weakness | 10028372 |
| Myalgia | 10028411 |
| Myoglobinuria | 10028629 |
| Myopathy | 10028641 |
| Myositis | 10028653 |
| Neuropathy peripheral | 10029331 |
| Optic ischaemic neuropathy | 10030924 |
| Peripheral motor neuropathy | 10034580 |
| Peripheral sensory neuropathy | 10034620 |
| Polymyositis | 10036102 |
| Polyneuropathy | 10036105 |
| Polyneuropathy alcoholic | 10036106 |
| Polyneuropathy idiopathic progressive | 10036111 |
| Polyneuropathy in malignant disease | 10036115 |
| Renal failure acute | 10038436 |
| Rhabdomyolysis | 10039020 |
| Sensory neuropathy hereditary | 10040037 |
| Skin depigmentation | 10040825 |
| Skin hyperpigmentation | 10040865 |
| Skin hypopigmentation | 10040868 |
| Splenomegaly | 10041660 |
| Toxic optic neuropathy | 10044245 |
| Uraemic neuropathy | 10046328 |
| Yellow skin | 10048245 |
| Sciatic nerve neuropathy | 10048950 |
| Cholestasis of pregnancy | 10049055 |
| Muscle fatigue | 10049565 |
| Ischaemic neuropathy | 10051307 |
| Neonatal cholestasis | 10056528 |
| Peripheral sensorimotor neuropathy | 10056673 |
| Subacute hepatic failure | 10056956 |
| Post cholecystectomy syndrome | 10057229 |
| Phagocytosis | 10057249 |
| Chronic hepatic failure | 10057573 |
| Chronic inflammatory demyelinating polyradiculoneuropathy | 10057645 |
| Ocular icterus | 10058117 |
| Optic neuropathy | 10061323 |
| Autonomic neuropathy | 10061666 |
| Demyelinating polyneuropathy | 10061811 |
| Lung infiltration malignant | 10062041 |
| Pigmentation disorder | 10062080 |
| Mononeuropathy | 10062203 |
| Congenital neuropathy | 10062346 |
| Mitochondrial hepatopathy | 10062938 |
| Polyneuropathy chronic | 10064135 |
| Macrophages increased | 10064665 |
| Obturator neuropathy | 10065292 |
| Multifocal motor neuropathy | 10065579 |
| HIV peripheral neuropathy | 10065681 |
| Cardiac autonomic neuropathy | 10066001 |
| Critical illness polyneuropathy | 10066336 |
| Acute polyneuropathy | 10066699 |
| Liver injury | 10067125 |
| Toxic neuropathy | 10067722 |

**Table S2** Definitions of network, taxonomic, and intrinsic features

| Feature name | Feature definition | Supplementary information |
| --- | --- | --- |
| Network feature |  |  |
| degree-prod | $X_{1}\left( i,j \right)=degree\left( i \right)\times degree\left( j \right)$ |  |
| degree-sum | $X_{2}\left( i,j \right)=degree\left( i \right)+degree\left( j \right)$ |  |
| degree-ratio | $X_{3}\left( i,j \right)=degree\left( i \right)/degree\left( j \right)$ |  |
| degree-absdiff | $X_{4}\left( i,j \right)=degree\left( i \right)-degree\left( j \right)$ |  |
| jaccard-drug-max | $X_{5}\left( i,j \right)=\max_{k\in N\left( j \right)-\left\{ i \right\}} \left\{ J\left( i,k \right) \right\}$ | $J\left( i,k \right)=\left\vert N\left( i \right)\cap N\left( k \right) \right\vert/\left\vert N\left( i \right)\cup N\left( k \right) \right\vert$ denotes the jaccard coefficient between the sets $N\left( j \right)$ and $N\left( k \right)$ |
| jaccard-drug-KL | $X_{6}\left( i,j \right)$: Kullback-Leibler (KL) distance between the distribution $D_{drug}\left( i,j \right)$ of the variable $J\left( j,k \right),k\in N\left( i \right)-\left\{ j \right\}$ and a reference distribution | The reference distribution was computed as the mean of distributions $D_{drug}\left( i,j \right)$ over the training edges $\left( i,j \right)$ |
| jaccard-ADE-max | $X_{7}\left( i,j \right)=\max_{k\in N\left( i \right)-\left\{ j \right\}} \left\{ J\left( j,k \right) \right\}$ |  |
| jaccard-ADE-KL | $X_{8}\left( i,j \right)$: KL distance between the distribution $D_{ADE}\left( i,j \right)$ of the variable $J\left( i,k \right),k\in N\left( j \right)-\left\{ i \right\}$ and a reference distribution | The reference distribution was computed as the mean of distributions $D_{ADE}\left( i,j \right)$ over the training edges $\left( i,j \right)$ |
| Taxonomic feature |  |  |
| atc-min | $X_{9}\left( i,j \right)=\min_{k\in N\left( j \right)-\left\{ i \right\}} \left\{ d_{ATC}\left( i,k \right) \right\}$ |  |
| atc-KL | $X_{10}\left( i,j \right)$: KL divergence between the distribution $D_{ATC}\left( i,j \right)$ of the variable $D_{ATC}\left( i,k \right) ,k\in N\left( j \right)-\left\{ i \right\}$and a reference distribution. | The reference distribution was computed as the mean of distributions $D_{ATC}\left( i,j \right)$ over the training edges (*i*, *j*) |
| meddra-min | $X_{11}\left( i,j \right)=\min_{k\in N\left( i \right)-\left\{ j \right\}} \left\{ d_{MedDRA}\left( j,k \right) \right\}$ |  |
| meddra-KL | $X_{12}\left( i,j \right)$: KL divergence between the distribution $D_{medDRA}\left( i,j \right)$ of the variable $D_{MedDRA}\left( j,k \right) ,k\in N\left( i \right)-\left\{ j \right\}$ and a reference distribution. | The reference distribution was computed as the mean of distributions $D_{MedDRA}\left( i,j \right)$ over the training edges (*i*, *j*) |
| Intrinsic feature |  |  |
| euclid-min | $X_{13}\left( i,j \right)=\min_{k\in N\left( j \right)-\left\{ i \right\}} \left\{ d_{INT}\left( i,k \right) \right\}$ |  |
| euclid-KL | $X_{14}\left( i,j \right)$: KL divergence $d_{INT}\left( i,j \right)$ between the distribution of the variable $d_{INT}\left( i,k \right),k\in N\left( j \right)-\left\{ i \right\}$  and a reference distribution. | The reference distribution was computed as the mean of distributions $d_{INT}\left( i,j \right)$ over the training edges (*i*, *j*) |

*Note*: Variable $i$ denotes a drug, variable $j$ denotes an ADE, $N\left( i \right)$ denotes the set of neighbors of node $i$, and $N\left( j \right)$ denotes the set of neighbors of node $j$. $d_{ATC}$ denotes the length of the shortest path between two drugs in the ATC taxonomy; $d_{\mathrm{MedDRA}}$ denotes the length of the shortest path between two ADEs in the MedDRA taxonomy, and $d_{INT}$ denotes the Euclidean distance between two drugs in the 17-dimensional intrinsic features space, subscript INT is an abbreviation for the intrinsic features.

**Table S3 (A)** Confusion matrices of IC_PNM_ and IC_PNM_ without controlling confounding bias using SIDER in table 2

|  | IC_PNM_ | | IC_PNM_ without controlling confounding bias | |
| --- | --- | --- | --- | --- |
|  | True signals | False signals | True signals | False signals |
| Detected | 315 | 52 | 306 | 54 |
| Undetected | 130 | 58 | 119 | 56 |

**Table S3 (B)** Confusion matrices of IC and EBGM using SIDER in table 2

|  | IC | | EBGM | | |
| --- | --- | --- | --- | --- | --- |
|  | True signals | False signals | | True signals | False signals |
| Detected | 238 | 31 | | 244 | 34 |
| Undetected | 207 | 79 | | 201 | 76 |

**Table S3 (C)** Confusion matrices of ROR and PRR using SIDER in table 2

|  | ROR | | PRR | |
| --- | --- | --- | --- | --- |
|  | True signals | False signals | True signals | False signals |
| Detected | 235 | 29 | 235 | 35 |
| Undetected | 211 | 81 | 211 | 75 |

**Table S4 (A)** Confusion matrices of IC_PNM_ and IC_PNM_ without controlling confounding bias using OMOP in table 2

|  | IC_PNM_ | | IC_PNM_ without controlling confounding bias | |
| --- | --- | --- | --- | --- |
|  | True signals | False signals | True signals | False signals |
| Detected | 53 | 42 | 53 | 43 |
| Undetected | 27 | 36 | 27 | 35 |

**Table S4 (B)** Confusion matrices of IC and EBGM using OMOP in table 2

|  | IC | | EBGM | |
| --- | --- | --- | --- | --- |
|  | True signals | False signals | True signals | False signals |
| Detected | 23 | 7 | 27 | 10 |
| Undetected | 57 | 71 | 53 | 68 |

**Table S4 (C)** Confusion matrices of ROR and PRR using OMOP in table 2

|  | ROR | | PRR | |
| --- | --- | --- | --- | --- |
|  | True signals | False signals | True signals | False signals |
| Detected | 37 | 28 | 37 | 28 |
| Undetected | 43 | 50 | 43 | 50 |

**Table S5 (A)** Confusion matrices of IC_PNM_ and IC_PNM_ without controlling confounding bias using SIDER in table 3

|  | IC_PNM_ | | IC_PNM_ without controlling confounding bias | |
| --- | --- | --- | --- | --- |
|  | True signals | False signals | True signals | False signals |
| Detected | 530 | 69 | 511 | 74 |
| Undetected | 85 | 81 | 104 | 76 |

**Table S5 (B)** Confusion matrices of IC and EBGM using SIDER in table 3

|  | IC | | EBGM | |
| --- | --- | --- | --- | --- |
|  | True signals | False signals | True signals | False signals |
| Detected | 320 | 31 | 370 | 34 |
| Undetected | 295 | 119 | 245 | 116 |

**Table S5 (C)** Confusion matrices of ROR and PRR using SIDER in table 3

|  | ROR | | PRR | |
| --- | --- | --- | --- | --- |
|  | True signals | False signals | True signals | False signals |
| Detected | 345 | 47 | 344 | 45 |
| Undetected | 270 | 103 | 271 | 105 |

**Table S6** Spearman correlation coefficient of different signal detection algorithms

|  | IC_PNM_ | EBGM | IC | ROR | PRR |
| --- | --- | --- | --- | --- | --- |
| IC_PNM_ | 1 | 0.6619 | 0.5039 | 0.1606 | 0.1602 |
| EBGM | 0.6619 | 1 | 0.8146 | 0.4975 | 0.4972 |
| IC | 0.5039 | 0.8146 | 1 | 0.8491 | 0.8489 |
| ROR | 0.1606 | 0.4975 | 0.8491 | 1 | 0.9999 |
| PRR | 0.1602 | 0.4972 | 0.8489 | 0.9999 | 1 |

**Table S7** Top-50 signals identified by Bayesian approaches can be validated in SIDER from FAERS 2004 to 2009

| EBGM | IC | IC_PNM_ |
| --- | --- | --- |
| Ibandronate-Myalgia | Quetiapine-Diabetic neuropathy | Infliximab-Rhabdomyolysis |
| Quetiapine-Neuropathy peripheral | Ofloxacin-Corneal deposits | Trazodone-Jaundice |
| Quetiapine-Diabetic neuropathy | Ziprasidone-Diabetic neuropathy | Trimethoprim-acute myocardial infarction |
| Acetaminophen-Acute hepatic failure | Gadopentetate Dimeglumine-Skin hyperpigmentation | Fluconazole-Muscle spasms |
| Acetaminophen-Liver injury | Timolol-Corneal deposits | Carbidopa-Renal failure acute |
| Risperidone-Diabetic neuropathy | Chlorpromazine-Diabetic neuropathy | Lopinavir-Rhabdomyolysis |
| **Telithromycin-Jaundice** | **Telithromycin-Jaundice** | Cyclophosphamide-Muscle spasms |
| Ibandronate-Muscle spasms | Thiothixene-Diabetic neuropathy | Fluconazole-Rhabdomyolysis |
| Lisinopril-Diabetic neuropathy | Risperidone-Diabetic neuropathy | Cytarabine-Myalgia |
| Zoledronate-Lymphadenopathy | Telithromycin-Ocular icterus | Adalimumab-Renal failure acute |
| Valdecoxib-acute myocardial infarction | Adenosine-Myositis | Ondansetron-Hepatic cirrhosis |
| Bevacizumab-Gastrointestinal haemorrhage | Olanzapine-Diabetic neuropathy | Hydroxychloroquine-Rhabdomyolysis |
| **Telithromycin-Liver function test abnormal** | Sevelamer-Skin hyperpigmentation | Ceftriaxone-Muscle spasms |
| Olanzapine-Diabetic neuropathy | Trifluoperazine-Diabetic neuropathy | Cyclophosphamide-Rhabdomyolysis |
| Tiotropium-Muscle spasms | Gadodiamide-Skin hyperpigmentation | Sertraline-Hepatic function abnormal |
| Natalizumab-Muscular weakness | Gadoversetamide-Skin hyperpigmentation | Pantoprazole-acute myocardial infarction |
| Calcium Gluconate-Muscle spasms | Benzatropine-Diabetic neuropathy | Cyclosporine-Hepatic cirrhosis |
| Fluconazole-Rhabdomyolysis | Haloperidol-Diabetic neuropathy | Fexofenadine-Hepatic failure |
| **Telithromycin-Hepatic failure** | Tretinoin-Chronic hepatic failure | Carbidopa-Jaundice |
| Natalizumab-Muscle spasms | Teprenone-Hepatic function abnormal | Lopinavir-Lung infiltration |
| Clonazepam-Diabetic neuropathy | Arsenic Trioxide-Hepatic function abnormal | Doxorubicin-Hepatomegaly |
| **Itraconazole-Hepatic function abnormal** | Glipizide-Diabetic neuropathy | Pravastatin-Lung infiltration |
| Ropinirole-Muscle spasms | Gemtuzumab Ozogamicin-Hyperbilirubinaemia | Ceftriaxone-Rhabdomyolysis |
| Fluoxetine-Diabetic neuropathy | Perphenazine-Diabetic neuropathy | Nefazodone-Lung infiltration |
| Sevelamer-Muscular weakness | Telithromycin-Splenomegaly | Diazepam-Jaundice |
| Glipizide-Diabetic neuropathy | Efavirenz-Biopsy liver abnormal | Lamivudine-acute myocardial infarction |
| Metoprolol-Diabetic neuropathy | **Telithromycin-Liver function test abnormal** | Ceftriaxone-Lung infiltration |
| Anastrozole-Myalgia | Vardenafil-Optic ischaemic neuropathy | Fluoxetine-Hepatic function abnormal |
| Insulin Aspart-Renal failure acute | Aripiprazole-Diabetic neuropathy | Ribavirin-acute myocardial infarction |
| Bupropion-Diabetic neuropathy | Micafungin-Hepatic function abnormal | Tenofovir-Gastrointestinal haemorrhage |
| Haloperidol-Neuropathy peripheral | Bortezomib-Peripheral motor neuropathy | Heparin-Myalgia |
| Glyburide-Diabetic neuropathy | Miglitol-Hepatic function abnormal | Etoposide-Muscle spasms |
| Calcitriol-Muscular weakness | Buspirone-Diabetic neuropathy | Propoxyphene-Hepatic failure |
| Pantoprazole-acute myocardial infarction | **Telithromycin-Hepatic failure** | Lactulose-Hepatic function abnormal |
| Ezetimibe-acute myocardial infarction | Bortezomib-Polyneuropathy | Clavulanate-Lung infiltration |
| Olmesartan-Hepatic function abnormal | Bortezomib-Peripheral sensory neuropathy | Ramipril-Hepatic function abnormal |
| Tacrolimus-Liver function test abnormal | Epoetin Alfa-Skin hyperpigmentation | Carvedilol-Jaundice |
| Olmesartan-Rhabdomyolysis | Zidovudine-Biopsy liver abnormal | Lactulose-Muscular weakness |
| Mycophenolate Mofetil-Hepatic function abnormal | Amphotericin B-Hyperbilirubinaemia | Loratadine-Hepatic function abnormal |
| Digoxin-Delirium | Calcium Acetate-Skin hyperpigmentation | Vincristine-Rhabdomyolysis |
| Aripiprazole-Diabetic neuropathy | Mirtazapine-Diabetic neuropathy | Cimetidine-Gastrointestinal haemorrhage |
| Rosiglitazone-Diabetic neuropathy | Cyclobenzaprine-Diabetic neuropathy | Amiodarone-Lymphadenopathy |
| Ezetimibe-Hepatic function abnormal | Ethambutol-Lymphadenopathy | Temazepam-Jaundice |
| Paroxetine-Diabetic neuropathy | Propofol-Myoglobinuria | Salmeterol-Hepatic function abnormal |
| Ziprasidone-Diabetic neuropathy | Phytonadione-Hyperbilirubinaemia | Gemcitabine-Rhabdomyolysis |
| Peginterferon Alfa-2A-Neuropathy peripheral | **Itraconazole-Hepatic function abnormal** | Tretinoin-Renal failure acute |
| Valsartan-Delirium | Cefuroxime-Corneal deposits | Doxorubicin-Muscle spasms |
| Temazepam-Neuropathy peripheral | Pindolol-Rhabdomyolysis | Metolazone-Muscle spasms |
| Insulin Aspart-Muscle spasms | Cefuroxime-Corneal opacity | Gatifloxacin-Rhabdomyolysis |
| Cyclophosphamide-Muscular weakness | Nevirapine-Biopsy liver abnormal | Ramipril-Lymphadenopathy |
| *Note*: Red font represents signals identified by different Bayesian methods using the SIDER data, and in which bolds are the same ADE signals detected. | | |

**Table S8** Top-50 signals identified by Bayesian approaches can be validated in SIDER from FAERS 2014 to 2019Q2

| EBGM | IC | IC_PNM_ |
| --- | --- | --- |
| Dasabuvir-Jaundice | Dasabuvir-Jaundice | Carvedilol-Hepatorenal syndrome |
| Mercaptopurine-Hepatocellular injury | Mercaptopurine-Hepatocellular injury | Metronidazole-Axonal neuropathy |
| Asunaprevir-Hepatic function abnormal | Dasabuvir-Ocular icterus | **Simvastatin-Hepatic necrosis** |
| **Posaconazole-Hepatic failure** | Ciclesonide-Liver function test abnormal | Furosemide-Axonal neuropathy |
| Nivolumab-Hepatic function abnormal | **Posaconazole-Hepatic failure** | **Ciprofloxacin-Jaundice cholestatic** |
| Ritonavir-Hepatic encephalopathy | Acebutolol-Hepatitis cholestatic | Citalopram-Hyperbilirubinaemia neonatal |
| Calcium Gluconate-Hepatic failure | Asunaprevir-Hepatic encephalopathy | Mercaptopurine-Hepatocellular injury |
| Ursodeoxycholate-Hepatic necrosis | Caspofungin-Hepatic necrosis | Lamotrigine-Hyperbilirubinaemia neonatal |
| Nivolumab-Hepatic failure | Nivolumab-Hepatic function abnormal | Ritonavir-Hepatic encephalopathy |
| **Ethionamide-Neuropathy peripheral** | Daptomycin-Hepatic encephalopathy | Cefazolin-Acute hepatic failure |
| Lenvatinib-Hepatic function abnormal | Rifampin-Hepatorenal syndrome | Oxaliplatin-Autonomic neuropathy |
| **Ciprofloxacin-Jaundice cholestatic** | **Ethionamide-Neuropathy peripheral** | Tretinoin-Skin hyperpigmentation |
| Carvedilol-Hepatorenal syndrome | Lenograstim-Hepatocellular injury | **Posaconazole-Hepatic failure** |
| Nimesulide-Liver injury | Aripiprazole-Hyperbilirubinaemia neonatal | Sertraline-Polyneuropathy |
| Foscarnet-Liver function test abnormal | Mirabegron-Muscle injury | Acetaminophen-Congenital neuropathy |
| Nivolumab-Hepatocellular injury | Tretinoin-Pigmentation disorder | Fentanyl-Hepatorenal syndrome |
| Mirabegron-Muscle injury | Sulfasalazine-Muscle fatigue | Cefazolin-Hepatic encephalopathy |
| Promazine-Hyperbilirubinaemia | **Roxithromycin-Jaundice** | Clavulanate-Hyperbilirubinaemia |
| **Goserelin-Hepatic function abnormal** | Palbociclib-Neuropathy peripheral | Fluorouracil-Liver injury |
| Dasabuvir-Cholestasis | Tocopherol-Cholestasis | Levothyroxine-Demyelinating polyneuropathy |
| Tiopronin-Myalgia | Ritonavir-Hepatic encephalopathy | Carvedilol-Polyneuropathy |
| Chlorambucil-Myositis | Remifentanil-Hepatocellular injury | Prednisolone-Skin depigmentation |
| **Ifosfamide-Polyneuropathy** | Amlodipine-Multifocal motor neuropathy | Diclofenac-Hepatic necrosis |
| Peramivir-Hepatic function abnormal | Dasabuvir-Cholestasis | Endopeptidases-Jaundice cholestatic |
| Levosimendan-Gastrointestinal haemorrhage | Ceftazidime-Hepatomegaly | Tacrolimus-Granulomatous liver disease |
| Levoleucovorin-Pigmentation disorder | Cycloadiphenine-Hepatic function abnormal | Lansoprazole-Peripheral motor neuropathy |
| **Cilazapril-Muscle spasms** | **Ciprofloxacin-Jaundice cholestatic** | Daptomycin-Hepatic encephalopathy |
| Remifentanil-Hepatocellular injury | Carvedilol-Hepatorenal syndrome | Diltiazem-Cholestasis |
| Tretinoin-Pigmentation disorder | Glycopyrronium-Rhabdomyolysis | Ciclesonide-Liver function test abnormal |
| **Gliclazide-Hepatic failure** | Norepinephrine-Delirium | Oxaliplatin-Skin hyperpigmentation |
| Evolocumab-Muscular weakness | **Ifosfamide-Polyneuropathy** | Dasabuvir-Jaundice |
| Sufentanil-Delirium | Thioguanine-Hyperbilirubinaemia | Metformin-Peripheral motor neuropathy |
| Chlorzoxazone-Muscle spasms | Ferrous Fumarate-Skin depigmentation | Aripiprazole-Hyperbilirubinaemia |
| Beclomethasone-Hepatic failure | Clotiazepam-Hepatocellular injury | Rivaroxaban-Muscle fatigue |
| Pemetrexed-Cholestasis | Cladribine-Hepatic failure | Ceftriaxone-Axonal neuropathy |
| Pembrolizumab-Lymphadenopathy | Lomustine-Hepatocellular injury | Tacrolimus-Toxic optic neuropathy |
| Topotecan-Neuropathy peripheral | Galantamine-Liver function test abnormal | Apixaban-Lymphadenopathy |
| Paritaprevir-Hepatic failure | Econazole-Hepatocellular injury | **Gliclazide-Hepatic failure** |
| Lactulose-Hepatitis cholestatic | Nivolumab-Hepatic failure | Granisetron-Hepatic failure |
| **Roxithromycin-Jaundice** | Levothyroxine-Hyperbilirubinaemia neonatal | Ranitidine-Jaundice cholestatic |
| Cobalamins-Muscle fatigue | **Goserelin-Hepatic function abnormal** | Irbesartan-Myopathy |
| Thiotepa-Delirium | Lenvatinib-Hepatic function abnormal | Gentamicin-Rhabdomyolysis |
| Prothionamide-Polyneuropathy | Magnesium-Splenomegaly | Mirabegron-Muscle injury |
| Midodrine-Hepatitis cholestatic | Sufentanil-Delirium | Bupropion-Jaundice cholestatic |
| Crotamiton-Hepatomegaly | Meropenem-Toxic neuropathy | Gliclazide-Hepatomegaly |
| Caspofungin-Hepatomegaly | Corticotropin-Skin hyperpigmentation | Diphenhydramine-Muscle fatigue |
| **Simvastatin-Hepatic necrosis** | **Cilazapril-Muscle spasms** | Bortezomib-Optic neuropathy |
| Pentazocine-Liver function test abnormal | Everolimus-Polyneuropathy | Linezolid-Toxic neuropathy |
| Galantamine-Liver function test abnormal | Ofloxacin-Polyneuropathy | Voriconazole-Hyperbilirubinaemia |
| Ifosfamide-Rhabdomyolysis | Endopeptidases-Jaundice | Citalopram-Demyelinating polyneuropathy |
| *Note*: Red font represents signals identified by different Bayesian methods using the SIDER data, and in which bolds are the same ADE signals detected. | | |

# Supplementary Figures

**Supplementary Figure 1.** Overview of PNM. First, three types of data were integrated, including FAERS data, taxonomic data (ATC code from DrugBank and MedDRA code), and intrinsic data (biochemical features of the drugs from PubChem). Second, the drug-ADE network was constructed based on the drug-ADE combinations from the training data. Third, the network features, taxonomic features, and intrinsic features were generated based on the drug-ADE network built upon the training data. Next, a logistic regression (LR) model was trained using the training set. Finally, its prediction performance was evaluated using the new drug-ADEs reported in the validation data set, and the probability of each drug-ADE association was generated by PNM.
